# Decision Support Methods for Finding Phenotype — Disorder Associations in the Bone Dysplasia Domain

**DOI:** 10.1371/journal.pone.0050614

**Published:** 2012-11-30

**Authors:** Razan Paul, Tudor Groza, Jane Hunter, Andreas Zankl

**Affiliations:** 1 School of ITEE, The University of Queensland, St. Lucia, Queensland, Australia; 2 Bone Dysplasia Research Group, UQ Centre for Clinical Research (UQCCR), The University of Queensland, Herston, Queensland, Australia; 3 Genetic Health Queensland, Royal Brisbane and Women’s Hospital, Herston, Queensland, Australia; University of Chicago, United States of America

## Abstract

A lack of mature domain knowledge and well established guidelines makes the medical diagnosis of skeletal dysplasias (a group of rare genetic disorders) a very complex process. Machine learning techniques can facilitate objective interpretation of medical observations for the purposes of decision support. However, building decision support models using such techniques is highly problematic in the context of rare genetic disorders, because it depends on access to mature domain knowledge. This paper describes an approach for developing a decision support model in medical domains that are underpinned by relatively sparse knowledge bases. We propose a solution that combines association rule mining with the Dempster-Shafer theory (DST) to compute probabilistic associations between sets of clinical features and disorders, which can then serve as support for medical decision making (e.g., diagnosis). We show, via experimental results, that our approach is able to provide meaningful outcomes even on small datasets with sparse distributions, in addition to outperforming other Machine Learning techniques and behaving slightly better than an initial diagnosis by a clinician.

## Introduction

Skeletal dysplasias are a heterogeneous group of genetic disorders affecting skeletal development. Currently, there are over 450 recognised bone dysplasias, classified into 40 groups. Patients with skeletal dysplasias can have complex medical issues including short stature, skeletal deformities, multiple fractures and neurological complications. However, since most skeletal dysplasias are very rare (<1∶10,000 births), data on clinical presentation, natural history and best management practices is sparse. Another reason for data sparseness is clinical variability, i.e., the small number of clinical features typically exhibited by patients from the large range of possible phenotypic and radiographic characteristics usually associated with these diseases. Due to the rarity of these conditions and the lack of mature domain knowledge, correct diagnosis is often very difficult. In addition, only a few centres worldwide have expertise in the diagnosis and management of these disorders. This is because, in the absence of defined guidelines, the diagnosis of new cases relies strictly on identifying similarities to past cases.

Medical decision support approaches, developed for particular diseases or groups of diseases (e.g., [1], [2], [3], [4], [5]), have demonstrated capabilities in assisting clinicians and researchers in research, as well as in the decision making process (e.g., diagnosis). Their main weakness is the need for well-documented domain knowledge built on generalised guidelines and supported by large-scale patient studies. Until now, this weakness has hindered the development of decision support methods in the bone dysplasia domain. Case-based reasoning [6] uses non-generalised evidences that do not guarantee correctness. Rule based systems [7] and fuzzy rule-based classification [8] use exact matching on rules that are built on mature and established domain knowledge – which is inapplicable in a domain that suffers from data sparseness. Neural networks [9] cannot provide justification for the resulting knowledge because they fuse all evidence into internal weights. In the skeletal dysplasia domain, however, justification is very important to both clinicians and researchers as it enables a better understanding of the underlying causal elements. Moreover, neural network approaches require large amounts of data for training.

Probabilities are useful when the knowledge required for inferencing or decision-making is not complete. Bayesian reasoning [10] is a widely used probability formalism, but it is problematic when applied in this domain due to the estimation of the prior and conditional probabilities. For example, in Bayesian reasoning, the probability of *Fruit = Apple* being given *Colour = Green*, i.e., Pr(*Fruit = Apple* | *Green*) would be zero if the training data set only contains *Fruit = Apple* in conjunction with *Colour = Red*. Laplace estimator is used to fix this issue by adding one to each count. This ensures that an attribute value that occurs zero times receives a probability that is nonzero. Although it works well in practice for many data sets, there is no specific reason for adding 1 to the counts. Dempster-Shafer theory (DST) [11], [12] is an alternative to representing probabilistic uncertainty mathematically. This is a potentially valuable tool in the decision making process when precise knowledge is missing [13]. An important aspect of this theory is the combination of evidence obtained from multiple sources with the computation of a degree of belief that takes into account all of the available evidence. Also, as opposed to Bayesian reasoning, DST does not require an estimation of the prior and conditional probabilities of the individual constituents of the set. Most of the previous applications of DST in medical decision support methods target bi-polar problems and require the existence of a domain knowledge base [14], [15].

In this paper, we describe an improved solution that minimises the impact of the data sparseness issue. Our approach is to analyse the underlying data characteristics to provide meaningful results from a set of inputs that are much smaller than the datasets usually required by decision support systems. The approach relies on mining association rules from patient descriptions (i.e., clinical features + diagnosis) and then using the Dempster-Shafer theory to produce probabilistic candidate rankings. Our goal is not to design a disease classifier (in the traditional sense), but rather to provide a data exploration mechanism to support clinicians in their decision making process. Hence we have chosen to produce probabilistic rankings associated with the underlying data justification.

## Materials and Methods

### Ethics Statement

The research and experiments presented in this paper have been conducted on anonymized data and with the consent of ESDN Clinical and Radiographic Management Group (ESDN-CRMG, http://www.esdn.org/).

**Figure 1:**
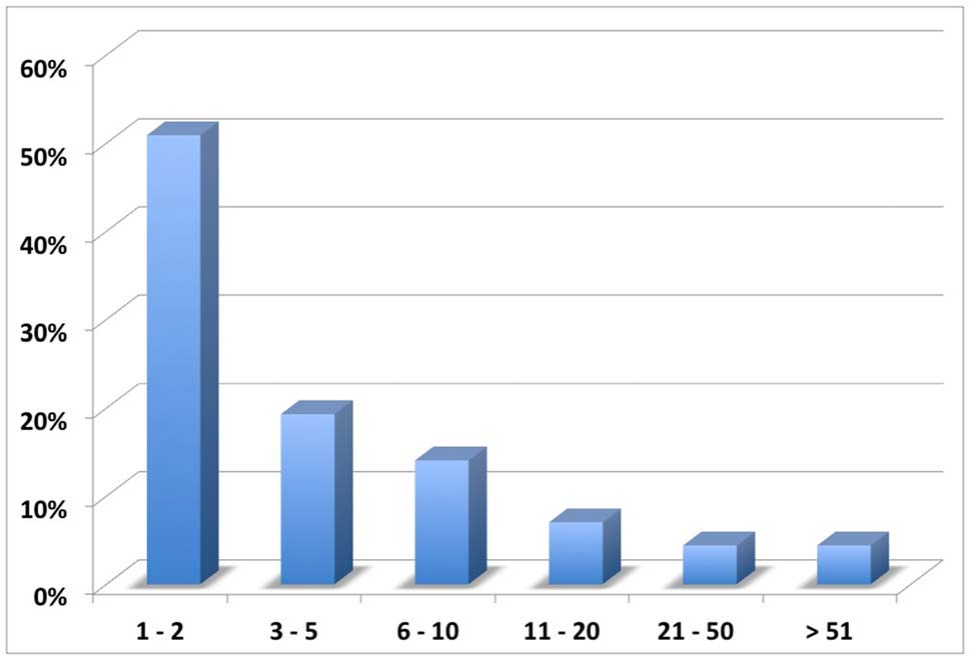
Relative distribution of dysplasia diagnoses according to different ranges of number of cases. More than 70% of the bone dysplasias present in the ESDN dataset have a very small number of cases (up to 5), while those that are well represented (i.e., over 50 cases) represent a mere fraction of the total number –4%.

### Data Characteristics

Data acquisition in the bone dysplasia domain is a challenging task. Different research groups around the world have, over time, built small patient registries that are neither open nor interoperable. In 2002, the European Skeletal Dysplasia Network (ESDN, http://www.esdn.org/) was created to alleviate, at least partly, the data sparseness issue, and at the same time to provide a collaborative environment to help with the diagnosis of skeletal dysplasias and to improve the information exchange between researchers. To date, ESDN has gathered over 1,200 patient cases, which have been discussed by its panel of experts.

The ESDN case workflow consists of three major steps: (1) a patient case is uploaded and an initial diagnosis is set by the original clinician that referred the case. Patient cases contain a free text clinical summary and associated X-Rays; (2) the panel of experts discusses the case until an agreement is reached; (3) the panel of experts recommends a diagnosis.

The approach described in this paper uses ESDN’s unique source of data for training and testing purposes. More specifically, we extracted clinical features from 1,281 patient clinical summaries and recorded the initial and final diagnoses. Among these cases, 744 have a bone dysplasia diagnosis (the remaining cases were not thought to be true bone dysplasias by the experts), and in total, there are 114 different skeletal dysplasias present. A brief analysis of the data reveals three important characteristics: (i) rareness, (ii) sparseness, and (iii) high dimensionality.

#### Rareness

Fig. 1 shows the relative distribution of diagnoses according to the number of cases. It can be observed that the vast majority of bone dysplasias (70%) have a very small number of cases (i.e., 1–2 or 3–5), while dysplasias well represented (i.e., over 50 cases) are a mere fraction of the total number (4%).

#### Sparseness

The coverage of clinical features in the patient clinical summaries provides a good indication of the data sparseness. The coverage of a single feature can be defined as the percentage of cases diagnosed with a particular dysplasia in which this phenotype is present. Table 1 shows a fragment of the coverage of some phenotypes, i.e., top three and bottom three. We exclude unique phenotypes occurring in a single case that has only this case as representative. The maximum coverage achieved is 50% (e.g., subglottic stenosis) and the minimum is 0.99% (e.g., immunodeficiency). Hence, the average coverage is 11.33%, with a median of 8% and a mode of 0.99%.

**Table 1:** Coverage of clinical and radiographic features in the ESDN dataset.

Clinical Feature	No. cases	Total diagnoses	Coverage (%)
Cystic hygroma	2	4	50
Subglottic stenosis	1	2	50
Cyanosis	1	2	50
Hypopigmentation of the skin	1	101	0.99
Immunodeficiency	1	101	0.99
Clumsiness	1	101	0.99

The table presents the top 3 and bottom 3 coverages. Coverage is computed by dividing the number of cases that contain the clinical feature by the total number of diagnoses denoting the bone dysplasias assigned to the cases. For example, *Cystic hygroma* appears in 2 of the total 4 cases diagnosed with Achondrogenesis type 1A.

#### High dimensionality

The skeletal dysplasia data emerging from the ESDN cases is characterised by a total of 602 unique clinical and radiographic features. Unfortunately, the distribution of these features across the different types of dysplasias is heavily skewed. Consequently, there are dysplasias characterised by 151 or 128 phenotypes (Spondyloepiphyseal dysplasia congenital – SEDC and Multiple epiphyseal dysplasia Autosomal Dominant - MED (AD), respectively), but there also are dysplasias with four or five phenotypes (e.g., Frontometaphyseal dysplasia or Neonatal Caffey disease). The average count of phenotypes per dysplasia is 21.58, with a median of 15 and a mode of 5.

### Proposed Classification Approach

We propose an approach that consists of three steps, discussed in the following sections: (i) data pre-processing, (ii) association rule extraction, and (iii) DST-based evidential reasoning. The data pre-processing phase extracts phenotypes from the patient clinical summaries using the Human Phenotype Ontology (HPO) [16] and structures the resulting annotations in a format suitable for input to the second step. The associate rule extraction uses a level wise search algorithm to infer evidences, which are then input to the evidential reasoning step. The confidence value of each association rule (evidence) is the conditional probability or the probabilistic uncertainty of the rule (the evidence). Based on a given set of clinical features, a set of suitable rules is selected from the rule base (that resulted from the rule extraction step). Finally, Dempster-Shafer theory is applied to compute the belief values for each candidate hypothesis that results from the selected set of rules.

#### Data pre-processing

As described above, patient clinical summaries in ESDN are represented in a free text format. In order to be able to use this data, we extracted patient phenotypes by annotating the text with corresponding terms from the Human Phenotype Ontology (HPO). In recent years, phenotype ontologies have been seen as an invaluable source of information, which can enrich and advance evolutionary and genetic databases [17]. HPO is currently the most comprehensive source of such information, comprising more than 10,000 terms organised in a hierarchical structure based on the anatomical localisation of the abnormality. The actual annotation process was performed using the National Centre for Biomedical Ontology (NCBO) Annotator [18], [19], an ontology-based web service for annotation of textual sources with biomedical concepts.

The annotation of a clinical summary results in a set of HPO terms, which are then transformed, together with the diagnosis, into a symbolic vector using a pre-computed domain dictionary. For example, *Short stature* is mapped to *S*_1_, *Cleft palate* to *S*_2_, Achondroplasia to *D*_1_, etc. The symbolic vector associated with each patient is used as input for the association rule mining process.

#### Association rule extraction

Association rule mining [20] discovers interesting associations within large sets of features, in principle, by considering the features that occur frequently together in a given dataset. For example, the association rule {Dwarfism} → {Achondroplasia} in a diagnosis context implies that if {Dwarfism} is present as a clinical feature in a patient, then the patient is likely to have Achondroplasia. Association rules provide knowledge in the form of probabilistic “if-then” statements. The head of the association rule (i.e., the “if” part) is called antecedent, while the body (i.e., the “then” part) is called consequent. The antecedent and consequent of an association rule are disjoint – they do not have any items in common. To express the uncertainty in association rules, two quantifiers are used: *support* and *confidence*. Support represents the number of transactions that include all items in the antecedent and consequent, and confidence is the ratio between the number of transactions that include all items in the consequent, as well as in the antecedent (namely, the support) and the number of transactions that include all items in the antecedent.
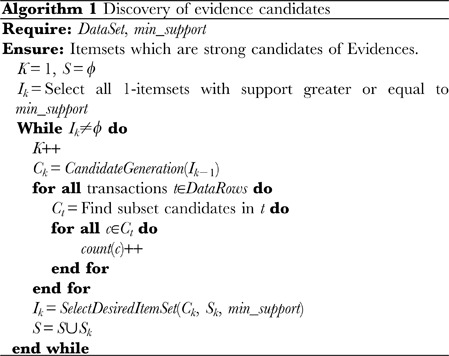


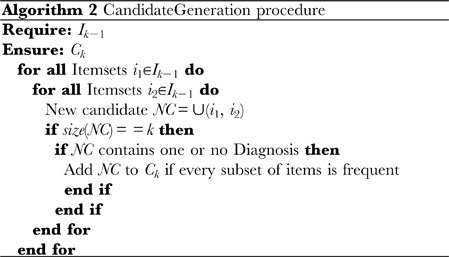


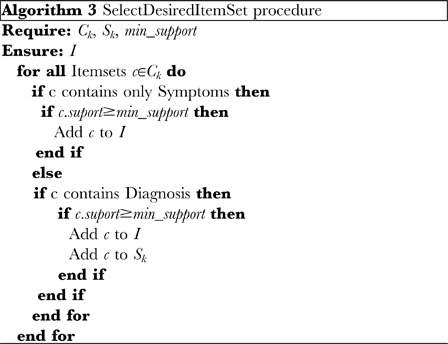


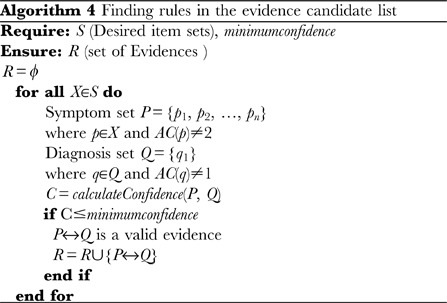



The actual extraction process consists of two parts: (1) discovering the trend in the instance data by finding a desired itemset using the adapted level wise search algorithm, and (2) finding rules (evidences) from the desired itemset. Alg. 0 details the trend discovery algorithm and Alg. 0 details the rule finding algorithm from desired itemsets. Following the discovery of the desired itemsets, these are partitioned into two components: a component containing the skeletal dysplasia and one containing the phenotypes. A boolean function that determines the type of a component is used to perform this classification. Subsequently, we calculate the confidence between the bone dysplasia component and the phenotype set of the rule (evidence) and use its value as the basic belief assignment of DST. For a better understanding, Appendix A within Supporting Information S1 contains a detailed example that illustrates this step.

We have used a relatively low minimum support of 2/N, where N is the total number of cases, because we are interested in extracting both frequent and rare associations. In addition, we need to take into account the fact that the data in our domain is rare. Every rule (evidence) contributes to the DST belief value of a proposition if the evidence is applicable to the proposition. Therefore, controlling the number of rules (evidences) using any minimum confidence threshold can bias the belief value and hence, the overall result. That is why we do not use this parameter to control the number of rules. We have also used a maximum itemset size of 10 as the computation cost increases exponentially with the itemset size in association rule mining.

#### DST-based evidential reasoning

Shafer [21] has expanded Dempster’s work [22] and proposed an evidence theory, currently referred to as the Dempster-Shafter Theory (DST). DST is a mathematical tool to express uncertain judgments based on all available evidences. The main advantages of DST over other probabilistic approaches are its ability to: [(i)] “model the narrowing of a hypothesis set with the accumulation of evidence” [23]; provide a representation for ignorance that is not uniformly distributed across all other alternative propositions; and avoid the Bayesian restriction according to which the commitment of belief to a hypothesis implies commitment of the remaining belief to its negation [23] – i.e., “Bayesian theory cannot distinguish between the lack of belief and disbelief” [21].

In addition, another important advantage of DST is that it allows evidence aggregation independent of the order of its gathering [23]. Finally, as opposed to Bayesian reasoning, Dempster-Shafer theory does not require an estimation of the prior and conditional probabilities of the individual constituents of the set. This is a potentially very important advantage in the decision making process where precise knowledge is missing. DST exposes four major functions: [(i)] Basic Belief Assignment (BBA); Combination of Evidence; Belief (Bel); and Plausibility An example that illustrates the use of these functions is presented in Appendix B within Supporting Information S1.

#### Basic Belief Assignment (BBA)

BBA expresses the degree of belief in a proposition and is the main information carrier. It does not refer to probability in the classical sense. BBA is assigned by making use of a mapping function (m) in order to define a mapping of the power set to the interval between 0 and 1, where the BBA of the null set is 0 and the summation of the BBA of all subsets of the power set is 1. The number 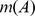 refers to the portion of total belief assigned exactly to proposition A. Mathematically, we can represent this in the following way:
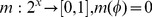
(1)

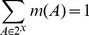
(2)where *x* is the universal set and 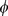 is the empty set.

#### Belief and plausibility functions

In order to infer meaningful information using BBA, it is imperative to impose a restriction on the total degree of belief in a proposition, as opposed to a single value of belief. This restriction on a proposition A is expressed by 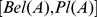 and it lies in the unit interval 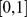, where 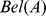 and 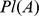 are given as:
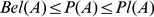
(3)

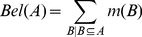
(4)

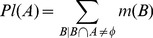
(5)


Here, *A* is a set of items and *B* is a subset of the set *A*. 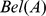 is the lower bound of the total degree of belief in a proposition *A* and is the sum of all BBAs of propositions that are fully included in proposition *A*. 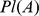 is the upper bound of the total degree of belief in a proposition *A* and is the sum of all BBAs of propositions whose intersection with proposition *A* is not empty. The set of all propositions that are of interest in DST is known as the frame of discernment.

#### The combination of evidence

The purpose of Combination of Evidence is to meaningfully summarize and simplify evidences, where the evidences are obtained from several independent knowledge sources over the same frame of discernment. In other words, this represents an evidence fusion. Evidence fusion refers to the combination of multiple sources to obtain an improved evidence by aggregating the complementary and/or redundant evidences. In widely used probability formalisms, probabilities are combined through the general multiplication operation. As DST is a generalisation of such probability formalisms, a more advanced multiplication rule is required for the combination of evidences. Dempster introduced Dempster’s rule of combination, which combines multiple evidences through their Basic Belief Assignments (*m*). These evidences are defined on the same frame of discernment and are independent. The rule is purely a conjunctive operation (AND) and results in a belief function based on conjunctive pooled evidence. If we obtain two masses 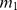 and 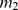, the combination (called the joint 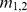) is calculated from two BBA’s 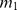 and 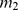 in the following manner:
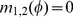
(6)

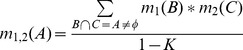
(7)where



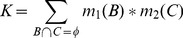
(8)Here, *B* and *C* are propositions from different knowledge sources, and *K* is a measure of the amount of conflict between the two masses 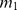 and 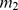. An example that illustrates the use of these functions is presented in Appendix B within Supporting Information S1. In practice, we model DST to express uncertain judgments about a set of conclusions in the presence of a set of observations, based on all available association rules (evidences).

#### Belief and plausibility function for a conclusion in the presence of a subset of observations

If we consider 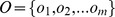 a set of observations and 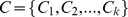 a set of conclusions, our goal is to find the overall belief of a conclusion in the presence of a subset of observations (the subset of observations that causes the conclusion). A proposition in our setting consists of a subset of observations *O*, named *A*, and an element of the conclusion set *C*, named 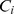. Hence, the proposition is 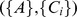. To achieve this, we have constrained the belief and plausibility function of Dempster-Shafer theory. We define the constrained belief, denoted by 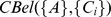, and the constrained plausibility, denoted by 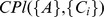, as follows:
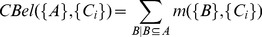
(9)with *A* being a subset of observations *O*, *B* a subset of *A* and 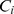 a conclusion in the set 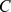. The belief 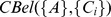 for the proposition 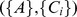 is defined as the sum of all masses of both subsets of *A* and 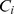




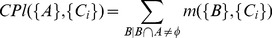
(10)

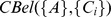
 is the lower bound of the total degree of belief of a conclusion in the presence of a subset of observations. It is the sum of all BBAs of those proposition subsets that have their observation elements fully included in proposition *A* and have the conclusion 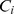. 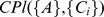 is the upper bound of the total degree of belief of a conclusion in the presence of a subset of observations. It is the sum of all BBAs of those proposition subsets that have observation elements included in proposition 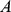 (i.e., their intersection with 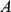 is not empty) and have the conclusion 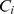.

If we consider a proposition to be a particular dysplasia in a patient, in the presence of a subset of observations, by calculating the belief and plausibility values, we can get the overall belief and plausibility degrees of the dysplasia in that patient. The soundness of the provided evidences is pivotal in medical decision-making. The plausibility value calculation takes into account the evidence that may exist or occur, while the belief value calculation relies on the evidence that must exist. Therefore, it is crucial to use only the belief value for decision-making and, hence avoid plausibility. Appendix C within Supporting Information S1 presents an example of the computation of these values extracted from the ESDN dataset.

### Other Classification Approaches Applied on the Dataset

Decision trees is a classification technique that maps observations about an item to conclusions about the item’s target value. Based on the training data, the decision tree learning algorithm constructs a tree-shaped model using inductive reasoning. To classify input data, each leaf of the tree represents a class label and the branches represent conjunctions of features that lead to those class labels. On the positive side of things, decision trees require no domain knowledge or parameter setting, and are easier to read and interpret. On the negative side of things, decision trees are unstable, in addition to being a hard classification model – i.e., it is limited to one output attribute. An improvement to the original algorithm has been brought by Quinlan, who proposed the Interactive Dichotomiser 3(ID3) [21]. ID3 uses information gain to select attributes to build the tree structure. Consequently, the process may result in a bias towards attributes with higher values.

Random forest is an ensemble classifier constructed from a number of decision trees [25]. Similar to the direct decision trees, random forest maps input data points to leafs in the tree, however, in this case, each tree in the forest assigns class label. The final class label for a point is assigned via voting, i.e., the class label that receives the most votes within the entire set of trees in the forest. For standard trees, nodes are split using the best split among all variables, whereas for random forests, nodes are split using the best among a subset of predictors randomly chosen at that node. Random forest has a number of advantages, such as the capability of handling high dimensional data, the effective method for estimating missing data, or the ability of generating an internal unbiased estimate of the generalisation error. Among the disadvantages, we can mention that it is, subject to the input data sets, prone to overfitting the data.

The naive Bayes classifier [26] is a classifier based on conditional probabilities and that is governed by the feature independence assumption. Despite this unrealistic assumption, the resulting classifier is remarkably successful in practice, often competing with much more sophisticated techniques [26]. It has proven results in most real-world applications, including text categorisation, medical decision support, and systems performance management [26]. The Naive-Bayes classifier features an extremely fast learning process that requires a single pass through the data. In addition, it usually requires a fairly small amount of training data, when compared to other approaches that achieve similar results. In our setting, since the skeletal dysplasia data is sparse, we have used the Laplace estimator to assign prior conditional probabilities when an attribute occurs zero times.

Medical decisions in the bone dysplasia domain are difficult as data is sparse. Subsequently, the representation of this sparse knowledge acts as a critical and differentiating factor between the methods that can be used as foundation for the decision support. A relevant example is the comparison between Bayesian and DST-based methods. A Bayesian method implies a precise value for prior probabilities. A critical difference, hence, between Bayesian and Dempster-Shafer-driven approaches lies in the representation of ignorance. In the computational phase of DST, all prior probabilities can be left unspecified, as opposed to a Bayesian method, which often requires an estimator to assign prior probabilities to random variables (e.g., by adding 1 where an attribute occurs zero times, so that its prior probability is non-zero).

Support Vector Machine (SVM) is a widely used machine learning technique, pioneered by Vapnik [27]. SVM trades off accuracy for the generalisation error. For pattern classification, SVM constructs a multidimensional hyperplane that optimally discriminates between two classes by maximising the margin between the two data clusters. To achieve a high discriminative power, SVM employs special nonlinear functions named kernels to convert the input data into a high-dimensional space.

The k-nearest neighbour algorithm (k-NN) [28] is a method for classifying an object by associating each data point of the object with its k-nearest neighbours. k-NN is unfortunately unable to provide a meaningful interpretation of high dimensional data. As the dimensionality of the data increases, the distance to the nearest point approaches the distance to the most distant point. k-NN also suffers from the data sparseness problem [29]. In the bone dysplasia domain, patients exhibit only a handful of clinical features from the entire range of possible phenotypic characteristics associated with bone dysplasias. Hence, each feature vector attached to a patient is very sparsely filled, while the missing features are different among the vectors. As a result, no similarity measures (e.g., Euclidean Distance, Hamming Distance, etc.) can provide meaningful interpretations to gauge similarity of samples in this domain. Support vector machines [27] also use similarity measures.

### Experimental Design

We compared our results against the initial diagnoses established by clinicians and against a set of well-known Machine Learning techniques. In order to provide the same experimental conditions, each approach used the same training and testing subsets. Some additional details associated with this comparative evaluation include: (i) Support Vector Machines (SVM) have been used with Sequential Minimal Optimisation for training and a polynomial kernel; (ii) the Naive Bayes classifier required the Laplace estimator to fix the absence of prior probability that occurs in sparse data; (iii) for k-NN we used k  = 3; (iv) in the case of decision trees, we have used the ID3 algorithm.

#### Dataset

In order to achieve realistic results Using machine learning methods, from the 114 existing types of dysplasias described in the ESDN dataset, we chose the types, each of which is represented by more than 20 patient cases. Table 2 displays the list of dysplasias (with > 20 patient cases) and the associated number of cases and other attributes. In total, 283 patient descriptions were considered (around 22% of the total cases). As shown in Table 2, the characteristics of the dataset we have chosen is similar to the general characteristics of the entire ESDN dataset.

**Table 2:** Characteristics of the skeletal dysplasias with more than 20 cases.

Diagnosis	Symbol	No. cases	Total features	Min	Max	Average	Max coverage	Largest common set coverage
Hypochondroplasia	*SD*_1_	22	69	1	15	5 (7.24%)	54.54%	27% (3)
SEDC	*SD*_2_	75	151	1	17	4.65 (3%)	40%	26% (2)
Pseudoachondroplasia	*SD*_3_	33	72	1	12	4.15 (5.76%)	57.77%	12% (3)
Cartilage-hair-hypoplasia	*SD*_4_	28	80	1	11	4.89 (6.11%)	46.42%	10% (4)
MED (AD)	*SD*_5_	101	128	1	20	3 (2.34%)	28.71%	19% (3)
rMED	*SD*_6_	24	59	1	13	3.87 (6.55%)	25%	25% (2)

The set of dysplasias used within our experiments follow, in principle, the general characteristics of the ESDN dataset. The average maximum coverage of phenotypes is around 43%, while the average largest common set (i.e., the set of phenotypes common to all cases diagnosed with a particular disorder) is around 20%.

#### Cross-validation

To estimate the predictive accuracy of diagnostic models, the dataset is usually split into two parts: a training set and a test set. The training set is used to establish the decision support model, while the test set is used to test the generalisation capability of the model. In this study, we applied a 5-fold cross-validation method to assess the model’s performance – training sets contain 80% of the cases and test sets contain 20% of the cases. As the dataset is very sparse, with some skeletal dysplasias having only 20 to 30 cases, 5-fold cross validation has the advantage that the resulting test sets are reasonably sized and have a fair distribution of clinical features for each bone dysplasia that we consider. The data listed in Table 2 was thus equally divided into five folds (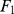 to 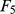) and five different sets of experiments were performed. Each of the 5 random partitions of the data serves as a test set for the diagnostic model trained with the remaining four partitions. The overall accuracy, precision and recall reported later in the paper represent an average across all 5 training set partitions.

The actual formulae used to compute these metrics are the following:
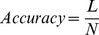
(11)

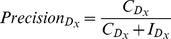
(12)

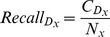
(13)

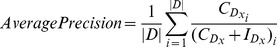
(14)

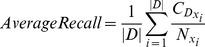
(15)where *N* is the total number of patient cases, *L* is the total number for correct predictions, *M* is the total number of incorrect predictions, and 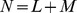. 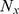 is the total number of dysplasia 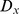 in *N*, 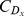 is total number of correct 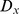 predictions, 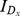 is total number of incorrect 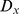 predictions.

## Results

In this section we discuss some experimental results we have achieved by applying our method to the ESDN dataset to examine its predictive capability in identifying classes of skeletal dysplasia. Table 3 displays the accuracy, precision and recall for each cross validation experiment with their corresponding intervals and mean. (each 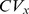 column refers to cross-validation on fold 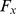). The accuracy rate across the five folds is fairly constant, resulting in a mean of 47.43%. Unlike accuracy, precision and recall are less stable, resulting in a fairly large interval of values with a mean of 39.34% for precision and 34.89% recall. To examine the prediction capability of the approach in a clinical setting, we compared the achieved accuracy against the initial diagnoses assigned to patient cases in ESDN. The comparison result, i.e., our average accuracy of 47.43% vs. the accuracy of the initial diagnoses –39.66%, shows that our approach outperforms these initial diagnoses by around 9%, which is an average decrease in error of 12%.

**Table 3:** Experimental results: Accuracy per cross-validation per fold.

	*CV*_1_	*CV*_2_	*CV*_3_	*CV*_4_	*CV*_5_	Interval	Mean (%)
Accuracy (%)	41.5	46	49.05	52.97	47.16	[41.5, 52.94]	47.43
Averageprecision (%)	31.08	51.63	51.61	41.69	20.71	[20.71, 51.63]	39.34
Averagerecall (%)	28.4	38.67	38.31	42.11	26.95	[26.95, 42.11]	34.89

## Discussion

In the previous section, we described the cross validation result of our approach, which has mean accuracy of 47.43% on the ESDN dataset and outperforms the clinicians’ initial diagnoses by around 9%. While the comparison of our approach against initial diagnosis is tempered because we have only considered six bone dysplasias, in practice, this level of improvement is realistic because for the initial diagnosis phase, on average only the 20 most common dysplasias are considered.

In the rest of this section, we provide a compare the results achieved by our solution against other Machine Learning approaches, and then we discuss our main findings, some related work and the limitations of our approach.

### Comparison Against other Machine Learning Approaches

For completeness purposes, we have compared our solution against the five most common Machine Learning approaches. Table 4 presents the experimental results. Overall, our approach outperforms all the other approaches. There are, nevertheless, some features that are worth noting. The Naive Bayes classifier performed well on 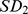, 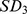 and 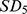, which is expected since it overfits the classes that provide more data at the expense of those that don’t. These dysplasias were the top three in terms of number of cases (see Table 2). Consequently, 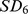 has 0% precision and 0% recall, while 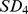 has 20% precision and 4% recall using the Naive Bayes classifier compared to 50% precision and 32% recall achieved by our approach. On a different note, our approach and SVM both performed uniformly across all classes, because they both generate an evenly distributed model – an overall best effort approach. Moreover, in the case of our solution, it can be clearly seen that precision and recall increase with the amount of data provided, reaching a maximum of 58.47% precision with only 101 patient cases provided for MED (AD).

**Table 4:** Experimental results: Overall comparative accuracy across all considered approaches.

	Our approach		Naive Bayes		SVM		Decision trees		Random forests		k-NN (K = 3)	
	P (%)	R (%)	P (%)	R (%)	P (%)	R (%)	P (%)	R (%)	P (%)	R (%)	P (%)	R (%)
*SD*_1_	6.67	5	20	5	11.66	10	13.16	15	15.54	15	10	5
*SD*_2_	47.71	70.12	53.66	34.72	38.12	40.46	29.78	32.98	36.98	33.08	31.92	38.34
*SD*_3_	42.41	27.78	41.66	9.16	36.42	25.26	25.18	26.94	25.8	24.98	5.84	11.66
*SD*_4_	50.67	32	20	4	33.08	32	44	24	21.42	20	20	4
*SD*_5_	58.47	59.43	41.94	97.64	47.07	64.14	52.88	45.52	50.64	59.2	45.04	62.52
*SD*_6_	30	15	0	0	40	15	21.68	31.66	10	10	0	0
Average recall (%)	34.89		25.08		31.14		29.35		27.55		20.25	
Average prec. (%)	39.34		29.54		34.38		31.11		26.8		18.8	
Accuracy rate (%)	47.43		44.12		41.08		33.83		36.89		34.23	

Our solution outperforms the five Machine Learning approaches we have considered within our experiments: around 4% more accuracy than Naive Bayes and around 6% more accuracy than SVM. Although Naive Bayes has performed very well, its results are boosted by overfitting the classes that had more data (e.g., ) at the expense of others, such as for which it achieved 0 precision and recall. Unlike Naive Bayes, our approach has performed fairly uniform and consistent across all classes.

Decision tree (ID3), on the other hand, prefers features with many values. The coverage of clinical features is an indicator of the data sparseness (i.e., features with a few or many values). The diagnoses built using a few examples, specifically SD1 (22), SD4 (28), SD6 (24), have an average coverage of 7.24%, 6.11% and 6.55%. These are greater than the average coverage of SD2 (75), SD3 (33) and SD5(101) –3%, 5.76%, 2.35% – which represent diagnoses with many examples. Consequently, we can infer that the SD1, SD4 and SD6 data is dense when compared to the SD2, SD3 and SD5 data. We believe that this is the reason behind the good performance achieved by the decision tree (ID3) approach.

Finally, when compared to the naive Bayes classifier, our approach shows improvements in the average accuracy from 44.12% to 47.43%, average precision from 29.54% to 39.34% and average recall from 25.08% to 34.89%. The justification of these results is the following: [(i)] unlike in Bayesian reasoning, ignorance is not represented as an uniform distribution in DST, and as opposed to DST, Bayesian reasoning uses Laplace estimator to estimate prior conditional probabilities when an attribute occurs zero times (sparse data). Both our approach, as well as the Bayesian classifier shows significant improvements in average accuracy, average precision and average recall over k-NN, which suffers from both the data sparseness and the high-dimensional data problems. A similar behaviour is observed also in the comparison against decision trees and random forests. As a final remark, while these performance indicators may seem low, they are in reality an improvement on the state of the art in decision support methods for rare disorders.

### Main Findings

We have presented an approach that combines association rule mining with the Dempster-Shafer theory (DST) to compute probabilistic associations between sets of clinical features and disorders. These can then serve as support for medical decision making (e.g., diagnosis). Experimental results show that the proposed approach is able to provide meaningful outcomes even on small datasets with sparse distributions. Moreover, the result shows that the approach can outperform other Machine Learning techniques and behaves slightly better than an initial diagnosis by a clinician. To test the accuracy of the approach, we have performed several experiments comparing human-mediated initial and final diagnoses, as well as outputs produced by other machine learning algorithms, in which we have treated our approach as a traditional classifier. The results show that we can achieve a top-1 accuracy of 47.43% (i.e., the accuracy calculated only via the candidate with the highest probability) by using disorder descriptions for 20 to more than 100 cases. This represents an increase in accuracy of around 7% when compared to the initial human-made diagnosis, and around 4% when compared to the next best machine learning approach.

### Related Work

The literature contains a few relevant approaches that are very similar to our general methodology. For example, the research presented in [30] employs association rule mining, Dempster’s rule of combination and pignistic approximation for the prediction of what else the customer is likely to buy. Once the rules are discovered, they use Dempster’s rule of combination to combine overlapping rules, followed by pignistic approximation for the final prediction. As opposed to our approach, the authors don’t use DST, per se, as they do not calculate any belief or plausibility values for prediction. However, when predictions systems need to calculate the joint probability of a set of items, where the items of the set are present in different association rules, it is imperative to use the DST believe value calculation. In their approach, the authors prune the overlapping antecedent rules as both the antecedent and consequent hold similar types of data (e.g., shopping items) in their discovered association rules. This is different to the way we have modelled the data, as in our case, the antecedent contains clinical features (observations), while the consequent holds the diagnosis (an action based on observation).

In [31], the proposed framework discovers interesting association rules and use the discovered rules for prediction. The rules get assigned basic belief values (BBAs) and are combined using Dempster’s rule of combination. Finally, maximum belief with non-overlapping interval strategy (maxBL) [32] or pignistic probability are used for prediction. In short, the authors use Dempster’s rule of combination to facilitate uncertainty management, without calculating belief and plausibility values as per DST.

The both of the above mentioned related works do not use DST to its full extent, but rather only Dempster’s rule of combination, in order to prune the number of rules. Instead of combining multiple evidences using a belief function, they choose the best evidence using a pignistic probability. Another significant difference is that they perform association rule mining for each query, whereas we generate evidences only once and use them as a knowledge base for all queries. The targeted domain of the first related work is market basket analysis. Like other classification algorithms, they do not measure accuracy to show the performance of their classification system. However, they measure precision and recall.The precision and recall is in the range of 40% to 60% and 50% to 60%, respectively, based on their synthetic dataset. The targeted domain of the second related work is sensor data analysis. The average accuracy of their proposed system is 47% based on the recorded sensor data. The characteristics of the data in our domain are significantly different than those of data in their domains. This makes it impossible to perform a fair performance comparison against them.

Finally, the research described in [33] employs evidence-based confidence, as opposed to traditional confidence, in the association rule mining, in order to describe evidences in a more sophisticated way. Their assumption is that some co-occurrences might be contingent in some datasets. The authors leverage DST to compute evidence-based confidence by introducing various kinds of ‘a posteriori’ pragmatic knowledge. Consequently, they focus on discovering sophisticated association rules, while we focus on using rules in conjunction with DST for classification purposes.

### Limitations

Two limitations are directly observable in the context of the research presented within this paper. Firstly, the ESDN dataset used within our experiments is based strictly on patients suspected to have a bone dysplasia. These patient cases are usually highly challenging, and thus, are submitted for evaluation by the ESDN panel of experts. Moreover, clinicians submitting cases to ESDN usually focus on providing only dysplasia-relevant data. Consequently, our experiments frame the underlying research question only to patient data that has a high probability to be associated with a bone dysplasia.

Secondly, our current analysis features only the combination of association rule mining with DST, and hence it does not reveal the results attributable individually to the association rule mining or to DST. As a remark, within our framework, we discover association rules that, by nature, cannot be used for classification (i.e., we do not discover classification rules similar to a branch in decision trees). To achieve this goal, such association rules require the conjunction with a prediction technique, e.g., voting [34], weighted voting [34], CMAR [35] or DST. In this paper, we have shown how to create an ensemble that combines association rules with DST. Future work will include also the evaluation of the other prediction techniques, in order to obtain an general view over the best classification method in our domain.

### Conclusion

The decision support method presented in this paper combines association rule mining with the Dempster-Shafer theory to produce probabilistic candidate phenotype–disease rankings. The experimental results we have presented demonstrate that, given a reasonable amount of data (considering the focus on rare diseases), our approach can outperform other Machine Learning techniques and behaves slightly better than an initial diagnosis by a clinician – which is often enough to guide further research on the case in the correct direction.

Future research will focus on the use of semantic relationships between patient phenotypes, expressed via the Human Phenotype Ontology, when mining association rules. For example, we will consider generalisations (i.e., *is-a* relationships) and partnonomies (i.e., *part-of* relationships) between the patient clinical and radiographic features. At the same time, we intend to incorporate and experiment with additional types of related data, such as, gene mutation data – in order to predict correlations between sets of phenotypes and gene mutations in the context of bone dysplasias.

## Supporting Information

Supporting Information S1 (PDF)

## References

[1] Ding J, Bashashati A, Roth A, Oloumi A, Tse K, et al.. (2011) Feature based classifiers for somatic mutation detection in tumour-normal paired sequencing data. Bioinformatics. 10.1093/bioinformatics/btr629 PMC3259434 22084253

[2] Tan AC, Naiman DQ, Xu L, Winslow RL, Geman D (2005) Simple decision rules for classifying human cancers from gene expression profiles. Bioinformatics 21: 2643–2644. 10.1093/bioinformatics/bti631 PMC1987374 16105897

[3] Hayward J, Alvarez SA, Ruiz C, Sullivan M, Tseng J, et al (2010) Machine learning of clinical performance in a pancreatic cancer database. Artificial Intelligence in Medicine 49: 187–195. 20483571 10.1016/j.artmed.2010.04.009

[4] Kopriva I, Filipovic M (2011) A mixture model with a reference-based automatic selection of com-ponents for disease classification from protein and/or gene expression levels. BMC Bioinformatics. 10.1186/1471-2105-12-496 PMC3292585 22208882

[5] Koehler S, Schulz MH, Krawitz P, Bauer S, Doelken S, et al (2009) Clinical diagnostics in human genetics with semantic similarity searches in ontologies. The American Journal of Human Genetics 85: 457–464. 19800049 10.1016/j.ajhg.2009.09.003 PMC2756558

[6] Begum S, Ahmed MU, Funk P, Xiong N, Folke M (2010) Case-Based Reasoning Systems in the Health Sciences: A Survey of Recent Trends and Developments. IEEE Transactions on Systems, Man, and Cybernetics, Part C: Applications and Reviews 41: 421–434.

[7] Hudson DL (2006) Medical Expert Systems. In: Akay M, editor, Wiley Encyclopedia of Biomedical Engineering, John Wiley and Sons.

[8] Gadaras I, Mikhailov L (2009) An interpretable fuzzy rule-based classification methodology for medical diagnosis. Artificial Intelligence in Medicine 47: 25–41. 19540096 10.1016/j.artmed.2009.05.003

[9] Chan KY, Ling SH, Dillon TS, Nguyen HT (2011) Diagnosis of hypoglycemic episodes using a neural network based rule discovery system. Expert Systems with Applications: An International Journal 38: 9799–9808.

[10] Martin J, Perez C, Muller P (2009) Bayesian robustness for decision making problems: Applications in medical contexts. International Journal of Approximate Reasoning 50: 315–323.

[11] Dempster AP (1967) Upper and Lower Probabilities Induced by a Multivalued Mapping. Annals of Mathematical Statistics 38: 325–339.

[12] Shafer G (1976) A mathematical theory of evidence. Princeton University Press.

[13] Yager RR, Liu L (2008) Classic Works of the Dempster-Shafer Theory of Belief Functions. Springer Verlag.

[14] Khatibi V, Montazer GA (2010) A fuzzy-evidential hybrid inference engine for coronary heart disease risk assessment. Expert Systems with Applications: An International Journal 37: 8536–8542.

[15] Straszecka E (2006) Combining uncertainty and imprecision in models of medical diagnosis. Infor-mation Sciences 176: 3026–3059.

[16] Robinson PN, Kohler S, Bauer S, Seelow D, Horn D, et al (2008) The Human Phenotype Ontology: A Tool for Annotating and Analyzing Human Hereditary Disease. The American Journal of Human Genetics 83: 610–615. 18950739 10.1016/j.ajhg.2008.09.017 PMC2668030

[17] Mabee PM, Ashburner M, Cronk Q, Gkoutos GV, Haendel M, et al (2007) Phenotype ontologies: the bridge between genomics and evolution. Trends in Ecology and Evolution 22: 345–350. 17416439 10.1016/j.tree.2007.03.013

[18] Jonquet C, Shah NH, Musen MA (2009) The Open Biomedical Annotator. In: Proceedings of the Summit on Translational Bioinformatics 2009. Thunder Bay, ON, US. pp 56–60. PMC3041576 21347171

[19] Roeder C, Jonquet C, Shah NH, Jr WAB, Verspoor K, et al (2010) A UIMA wrapper for the NCBO annotator. Bioinformatics 26: 1800–1801. 20505005 10.1093/bioinformatics/btq250 PMC2894505

[20] Agrawal R, Srikant R (1994) Fast Algorithms for Mining Association Rules in Large Databases. In: Proceedings of the Proceedings of the 20th International Conference on Very Large Data Bases. Santiago de Chile, Chile. pp 487–499.

[21] Shafer G (1976) A mathematical theory of evidence, volume 76. Princeton university press Prince-ton.

[22] Dempster A (1967) Upper and lower probabilities induced by a multivalued mapping. The Annals of Mathematical Statistics : 325–339.

[23] Gordon J, Shortliffe E (1984) The dempster-shafer theory of evidence. Rule-Based Expert Systems: The MYCIN Experiments of the Stanford Heuristic Programming Project 3: 832–838.

[24] Quinlan J (1986) Induction of decision trees. Machine learning 1: 81–106.

[25] Breiman L (2001) Random forests. Machine learning 45: 5–32.

[26] Rish I (2001) An empirical study of the naive bayes classifier. In: IJCAI 2001 workshop on empirical methods in artificial intelligence. volume 3, 41–46.

[27] Vapnik V (1995) The Nature of Statistical Learning Theory. Springer-Verlag.

[28] Gan Y, Guan J, Zhou S (2009) A pattern-based nearest neighbor search approach for promoter prediction using DNA structural profiles. Bioinformatics 25: 2006–2012. 19515962 10.1093/bioinformatics/btp359

[29] Grcar M, Mladenic D, Fortuna B, Grobelnik M (2006) Data Sparsity Issues in the Collaborative Filtering Framework. In: Proceedings of the 7th International Workshop on Knowledge Discovery on the Web. Chicago, IL, US. pp 58–76.

[30] Wickramaratna K, Kubat M, Premaratne K (2009) Predicting missing items in shopping carts. Knowledge and Data Engineering, IEEE Transactions on 21: 985–998.

[31] Hewawasam KKR, Premaratne K, Shyu ML (2007) Rule mining and classification in a situation assessment application: A belief-theoretic approach for handling data imperfections. Systems, Man, and Cybernetics, Part B: Cybernetics, IEEE Transactions on 37: 1446–1459. 10.1109/tsmcb.2007.903536 18179065

[32] Bloch I (1996) Some aspects of dempster-shafer evidence theory for classification of multi-modality medical images taking partial volume effect into account. Pattern Recognition Letters 17: 905–919.

[33] Murai T, Kudo Y, Sato Y (2003) Association rules and dempster-shafer theory of evidence. In: Discovery Science. Springer. pp 377–384.

[34] Azevedo P, Jorge A (2007) Comparing rule measures for predictive association rules. Machine Learning: ECML 2007 510–517.

[35] Li W, Han J, Pei J (2001) Cmar: Accurate and efficient classification based on multiple class-association rules. In: Data Mining, 2001. ICDM 2001, Proceedings IEEE International Conference on. IEEE. pp 369–376.

