# Supplementary material for: Decision Support Methods for Finding Phenotype — Disorder Associations in the Bone Dysplasia Domain
Source: PLoS One. 2012 Nov 30;7(11):e50614. doi: 10.1371/journal.pone.0050614 (PMC3511538; doi:10.1371/journal.pone.0050614)
Supplement: Supporting Information S1 — (PDF) [file pone.0050614.s001.pdf]

Supporting Information

Appendix A. Example of association rule mining output

Let us consider 3 symptoms and 2 skeletal Dysplasias defined in the following:

- A1 = Short stature
- A2 = Macrocephaly
- A3 = Osteoarthritis
- D1 = Hajdu-Cheney syndrome
- D2 = Diastrophic dysplasia

Let us also consider the following 4 patient cases:

- Patient 1 - {A1, A2, D1}
- Patient 2 - {A1, A2, D1}
- Patient 3 - {A1, A3, D2}
- Patient 4 - {A1, A3, D2}

Assuming minimum support 50% and minimum confidence 80%, the rule extraction algorithm presented in the paper will generate the following rules:

{A2} -> {D1} with confidence 1

{A3} -> {D2} with confidence 1

{A1, A2} -> {D1} with confidence 1

{A1, A3} -> {D2} with confidence 1

<u>Appendix B.</u> Example of Basic Belief Assignment and Belief computation in Dempster-Shafer theory.

Let us consider a skeletal dysplasia patient examined by two clinicians. Let us assume the first clinician assigns a 70% certainty for the patient to have PSACH or SEDC and the second clinician assigns a 50% certainty for the patient to have SEDC or MED . The frame of discernment will hence be: Y= {PSACH, SEDC, MED}.

The assumption of the first clinician leads to a BBA m_1 of 0.7 given the focal element {PSACH, SEDC}, i.e., m_1({PSACH, SEDC}) = 0.7. Provided that we have no additional about the remaining probability, this is allocated to the whole frame of the discernment, i.e., m_1({PSACH, SEDC, MED}) = 0.3. Similarly, the assumption of the second clinician assumption leads to a BBA m_2({SEDC, MED }) = 0.5, the remaining probability allocated to the whole the frame of the discernment m_2({PSACH, SEDC, MED}) = 0.5.

We can combine the BBA's from these two different sources to express our overall belief using Dempster's rule of combination. The result of combining two BBAs of sets B and C, where m_1({B}) = M1 and m_1({C}) = M2 is the BBA of the intersection of sets B and C. This BBA value is the product of M1 and M2 as the value of K is zero due to the fact that there is no set where the intersection is null.

$$m_3(\{SEDC, MED\}) = m_1(\{PSACH, SEDC, MED\}) \times m_2(\{SEDC, MED\}) = 0.3 \times 0.5 = 0.15$$

	m_1({PSACH, SEDC })=0.7	m_1({PSACH, SEDC, MED })=0.3
m_2({SEDC, MED })=0.5	m_3({SEDC})=0.35	m_3({SEDC, MED })=0.15
m_2({PSACH, SEDC, MED })=0.5	m_3({PSACH, SEDC })=0.35	m_3({PSACH, SEDC, MED })=0.15

Combination of the two evidences

From the combination of the two evidences we can express our overall belief in any set. The belief in any set is calculated by adding all BBAs of all the subsets of that set. For example:

Bel({PSACH, SEDC }) = m_3({ PSACH }) + m_3({ SEDC }) + m_3({PSACH, SEDC })
= 0 + 0.35 + 0.35 = 0.7

Bel({SEDC, MED }) = m_3({ SEDC }) + m_3({ MED }) + m_3({SEDC, MED })
= 0.35 + 0 + 0.15 = 0.5

Appendix C. **Example of Constrained Belief computation**

Aim: Find the diagnosis for an undiagnosed skeletal dysplasia patient with following symptoms: Short Stature (A_1) and Osteoarthritis (A_2).

Let us consider the following set of hypotheses and conclusions for the dysplasia domain:

- A_1 = Short stature
- A_2 = Osteoarthritis
- A_3 = Short legs
- A_4 = Short arms
- A_5 = Large head
- D_1 = Diastrophic dysplasia
- D_2 = Achondroplasia

Let's consider the following evidences in the rule set:

$\{A_1, A_3\} \rightarrow D_1$ with confidence 0.7

$\{A_2, A_4\} \rightarrow D_1$ with confidence 0.8

$\{A_1, A_3\} \rightarrow D_2$ with confidence 0.7

$\{A_2, A_4\} \rightarrow D_2$ with confidence 0.6

$\{A_3, A_4\} \rightarrow D_2$ with confidence 0.65

$\{A_3, A_5\} \rightarrow D_2$ with confidence 0.3

$\{A_4, A_5\} \rightarrow D_2$ with confidence 0.2

Solution

Step 1: Select the suitable evidences based on the symptoms of the patient (A1, A2):

$\{A_1, A_3\} \rightarrow D_1$ with confidence 0.7 (1)

$\{A_2, A_4\} \rightarrow D_1$ with confidence 0.8 (2)

$\{A_1, A_3\} \rightarrow D_2$ with confidence 0.7 (3)

$\{A_2, A_4\} \rightarrow D_2$ with confidence 0.6 (4)

Step 2: We select the candidate hypothesis based on the selected rules. These are: D1, D2. Hence the propositions of interest in the diagnosis of the patient will be:

- $\{A_1, A_2, D_1\}$
- $\{A_1, A_2, D_2\}$

Step 3: We perform belief value calculation for each candidate hypothesis.

Belief value calculation for D_1:

The hypothesis elements of the patient for conclusion D_1: A_1, A_2, A_3, A_4, D_1.

From (1), m_1 ({A_1, A_3, D1}) = 0.7 (from this, we get m_1 ({A_1, A_2, A_3, A4, D_1}) = 0.3}

From (2), m_2 ({A_2, A_4, D1}) = 0.8 (from this, we get m_2 ({A_1, A_2, A_3, A4, D_1}) = 0.2}

Combination of two pieces of evidence (m_1, m_2) through the Dempster's rule of combination and here K= 0 as there is no set where the intersection is null:

	$m_2\{A_2, A_4, D_1\}= 0.8$	$m_2\{A_1, A_2, A_3, A_4, D_1\} = 0.2$
$m_1\{A_1, A_3, D_1\}= 0.7$	$m_3\{D_1\} = 0.56$	$m_3\{A_1, A_3, D_1\} = 0.14$
$m_1\{A_1, A_2, A_3, A_4, D_1\} = 0.3$	$m_3\{A_2, A_4, D_1\}= 0.24$	$m_3\{A_1, A_2 , A_3, A_4, D_1\} = 0.06$

From the combination of these evidences, we can calculate overall belief associated with Dysplasia D_1.

$Bel(\{A_1, A_2, D_1\}) = m_3(\{D_1\}) + m_3(\{A_1, D_1\}) + m_3(\{A_2, D_1\}) + m_3(\{A_1, A_2, D_1\})$

$= 0.56 + 0 + 0 + 0$

$= 0.56$

Belief value calculation for D_2:

The hypothesis elements of the patient for conclusion D_2: A_1, A_2, $A_{3,}$ A_4, D_2.

From (3), m_1 $\{A_1, A_3, D_2\} = 0.7$ (from this, we get m_1 $\{A_1, A_2, A3, A_4, D_2\} = 0.3\}$

From (4), m_2 $\{A_2, A_4, D_2\} = 0.6$ (from this, we get m_2 $\{A_1, A_2, A_3, A4, D_2\} = 0.4\}$

Combination of two pieces of evidence (m_1, m_2) through the Dempster's rule of combination and here K= 0 as well.

	$m_2\{A_2, A_4, D_2 \}= 0.6$	$m_2\{A_1, A_2, A_3, A_4, D_2 \} = 0.4$
$m_1\{A_1, A_3, D_2 \}= 0.7$	$m_4\{ D_2 \} = 0.42$	$m_4\{A_1, A_3, D_2 \} = 0.28$
$m_1\{A_1, A_2, A_3, A_4, D_2 \} = 0.3$	$m_4\{A_2,A_4, D_2 \} = 0.18$	$m_4\{A_1, A_2, A_3, A_4, D_2 \} = 0.12$

$Bel(\{A_1, A_2, D_2\}) = m_3(\{D_2\}) + m_3(\{A_1,D_2\}) + m_3(\{A_2,D_2\}) + m_3(\{A_1,A_2,D_2\})$

$= 0.42 + 0 + 0 + 0$

$= 0.42$

Step 4: We rank the belief values and choose the highest as the best candidate. Considering the calculations above, the ranking of the belief values is: D_1, D_2.
